# Vaccine Inoculation

**Published:** 1802-12-01

**Authors:** 


### VACCINE INOCULATION.

THE following extract of a letter from Dr. Brandreth, of Liverpool, to Dr. Marfhall, contains a refpectable teftimonial in favour of Vaccine Inoculation; fuch a teftimonial, as it would be injuftice to the public to withold, while a veftige of that cruel peftilence, the fmall-pox, remains.

"No circumftance of importance relative to the cow-pox, has occurred in this neighbourhood. All the medical practitioners here, who ftand high in character, recommend, and have adopted the Inoculation of it, with full confidence of its being an effectual fecurity againft the fmall-pox. In the adjacent towns of Ormfkirk, Prefcot, and St. Helens, it has been extenfively employed; nor can there be any doubt that it will fhortly become univerfal. The fafety with which it may be performed at any age, and under every ftate of health, (its efficiency being once eftablifhed, which indeed is, at prefent, indifputably the cafe) muft overcome every obftacle, and filence the cavils of ignorance, prejudice, and malevolence. It muft prevail, and cannot fail to be efteemed the moft valuable bleffing that ages and centuries have beftowed on the human race.

"But it is wholly unneceffary to repeat the advantages which muft accrue from this difcovery, fince they have already been fo ably illuftrated by Mr. Ring, whofe treatife on this fubject is a mafter-piece, written with zeal, candour, and great knowledge of the fubject.

"The mafs of evidence he has produced in its favour, would convert an infidel. He has, in the moft confpicuous and fatisfactory manner, proved, that a perfon who has once had the real cow-pox, remains, for ever, infufceptible of the fmall-pox; and I peculiarly admire the ingenuity and fuccefs with which he has detected the fources of unfavourable reports, and laid before his readers, in the compafs of a moderate volume, all the knowledge, and an analytical review of all the publications on this interefting fubject.—I can add nothing ufeful.

"Dr. Jenner, to whom the world is infinitely indebted for the candid and expeditious manner in which he communicated his knowledge on this fubject, has my moft fincere thanks, and ardent wifhes, that the public remuneration may be ample and honourable. You will oblige me greatly, by making my beft regards acceptable to him. He has my beft wifhes for his happinefs and fuccefs."

